# Quantification of Protein “Biomarkers” in Wheat-Based Food Systems: Dealing with Process-Related Issues

**DOI:** 10.3390/molecules27092637

**Published:** 2022-04-20

**Authors:** Mauro Marengo, Aristodemo Carpen, Gianfranco Mamone, Pasquale Ferranti, Stefania Iametti

**Affiliations:** 1 Dipartimento di Scienza e Tecnologia del Farmaco, Università degli Studi di Torino, Via Giuria 9, 10125 Torino, Italy; mauro.marengo@unito.it; 2 Department of Food, Environmental and Nutritional Sciences (DeFENS), Università degli Studi di Milano, via G. Celoria 2, 20133 Milan, Italy; aristodemo.carpen@unimi.it; 3 Istituto di Scienze dell’Alimentazione, Consiglio Nazionale delle Ricerche, Via Roma, 64, 83100 Avellino, Italy; gianfranco.mamone@isa.cnr.it; 4 Dipartimento di Agraria, Università degli Studi di Napoli “Federico II”, Via Università 100, 80055 Portici, Italy; pasquale.ferranti@unina.it

**Keywords:** food proteins, biomarkers, gluten, ovalbumin, lectin, pasta, egg, whole grain, immunochemistry

## Abstract

Selected food proteins may represent suitable markers for assessing either the presence/absence of specific food ingredients or the type and intensity of food processes. A fundamental step in the quantification of any protein marker is choosing a proper protocol for solubilizing the protein of interest. This step is particularly critical in the case of solid foods and when the protein analyte is prone to undergo intermolecular disulfide exchange reactions with itself or with other protein components in the system as a consequence of process-induced unfolding. In this frame, gluten-based systems represent matrices where a protein network is present and the biomarker proteins may be either linked to other components of the network or trapped into the network itself. The protein biomarkers considered here were wheat gluten toxic sequences for coeliac (QQPFP, R5), wheat germ agglutinin (WGA), and chicken egg ovalbumin (OVA). These proteins were considered here in the frame of three different cases dealing with processes different in nature and severity. Results from individual cases are commented as for: (1) the molecular basis of the observed behavior of the protein; (2) the design of procedure aimed at improving the recovery of the protein biomarker in a form suitable for reliable identification and quantification; (3) a critical analysis of the difficulties associated with the plain transfer of an analytical protocol from one product/process to another. Proper respect for the indications provided by the studies exemplified in this study may prevent coarse errors in assays and vane attempts at estimating the efficacy of a given treatment under a given set of conditions. The cases presented here also indicate that recovery of a protein analyte often does not depend in a linear fashion on the intensity of the applied treatment, so that caution must be exerted when attributing predictive value to the results of a particular study.

## 1. Introduction

In a broad range of foods, appropriately selected protein components may provide a convenient, fast, and reliable way of investigating both the origin of the raw materials and/or the nature and intensity of the treatments used for increasing the stability of the starting materials or for processing them into shelf-ready and shelf-stable foods. Analytical approaches may include protein isolation, identification, and quantification, typically carried out by separation techniques that often involve steps that do not require maintaining the original structure of the protein in its intact, native form or exploiting changes (solubility, or adduct formation, among others) that are induced by physical, chemical, or enzymatic treatments. In view of their practical relevance to the food industry at large, an extremely broad array of methodologies has been deployed to detect and quantitate potential biomarkers, which can be essentially grouped in three main classes: (i) DNA-based; (ii) mass spectrometry-based (MS-based); (iii) bio-based.

DNA-based approaches rely on quantitative PCR and offer a number of advantages, ranging from high sensitivity to relatively low cost, and from minimal sample size to high specificity. However, they can show the presence of a given DNA when a protein is no longer present. Conversely, a recognizable protein biomarker may be present despite the absence of DNA from the species under scrutiny. The latter case is much worse than the former one—in particular, when health-relevant proteins are involved (say, food allergens or gluten, among others) [1].

MS-based approaches typically nowadays rely on highly specialized (and expensive) combinations of chromatography and mass spectrometry that use extremely minute amounts of sample and offer amazing selectivity and sensitivity. However, there are several issues with the quantitative analysis of MS results, as well as with some aspects of sample preparation, including recovery issues (as discussed later in this report) and issues concerning the residual biological activity of a given biomarker. Indeed, the conditions and the procedures used for sample preparation may involve chemical modification of the protein (e.g., alkylation of cysteine thiols or removal of some post-translational modification) and almost invariably call for a selective proteolysis step [2].

Measurements based on biological activity offer the advantage of high sensitivity and specificity, along with high throughput and—in most cases—low unit cost. Biological activity measurements can rely on the catalytic activity of the protein(s) under scrutiny, but most often exploit high-specificity protein–protein or protein–ligand interactions. Whereas the applications of protein–ligand interactions in the general area of food analysis are limited, the strong and very specific antigen–antibody interaction has emerged as a “de facto” standard in a number of food-relevant cases, with a plethora of commercially available antibodies and immunochemical kits [1,3].

All the approaches mentioned above can be applied rather straightforwardly to food matrices where the proteins under investigation are present in a soluble or easy-to-solubilize form (for example: milk, whey, or fermented dairy foods). On the contrary, the application of immunochemical approaches (and of other approaches as well) remains a problem in complex matrices where a protein network is present and the target proteins may be either linked to other components of the network (including molecules of the same species under scrutiny) or physically entangled within a process-generated polymeric network [4]. In both cases, reliable quantitation calls for sample treatments capable of breaking down the network while allowing the protein to retain its ability to be recognized.

In this study, we used unpublished data to present and discuss three cases of detection of specific protein biomarkers in gluten-based systems of different composition and/or at different processing stages. The gluten network has a fundamental role in defining the texture of many staple foods, such as pasta or bread, and originates from the permanent formation of aggregates among the water-insoluble gliadins and glutelins. These aggregates are stabilized by both non-covalent hydrophobic interactions and intermolecular disulfide exchange among the involved proteins [5].

The protein biomarkers considered here are wheat gluten toxic sequences for coeliac (henceforth, QQPFP (R5)), wheat germ agglutinin (WGA), and chicken egg ovalbumin (OVA). All are common in food formulations and share some chemical and physical features. All of them are monomeric in their native form, as found in raw materials, and have most (OVA) or all of their cysteine residues (gliadin and WGA) involved in intramolecular disulfide bonds. One of them (OVA) is glycosylated and phosphorylated on a number of possible different sites and contains a single disulfide bridge (the remaining four cysteine residues have free thiols), whereas gliadin and WGA do not undergo post-translational modifications other than formation of intramolecular disulfide bridges involving all the cysteine residues in the protein. Gliadins contain sequences reportedly relevant to celiac individuals, OVA is listed as a common food allergen, and WGA is a lectin. All have structures that may undergo process-dependent modifications that depend on the nature and intensity of the process and may lead to intermolecular disulfide exchange reactions with other protein components of the system to give a stable covalent network.

Processes presented in this study address model and real systems in which wheat proteins are the only or the prevalent component. The processes under scrutiny cover the range from the simplest ones (such as industrial pasta making) to others that may be placed at the opposite range of the complexity/cost spectrum in the food industry (such as treatment at high hydrostatic pressure). The focus of this report will be on: (1) the molecular basis of the observed behavior in individual process/protein cases; (2) the design of specific procedures required to have the protein biomarker in a form suitable for reliable identification and quantification; (3) a critical analysis of the difficulties associated with transfer of analytical protocols from one product/process to another.

## 2. Results and Discussion

### 2.1. Detection of Gliadin in Processed Dough

Quantification of gluten protein in food is fundamental for assessing gluten levels, a parameter that is of paramount relevance for gluten-intolerant people [6]. All food processes in wheat systems result in structural protein modification—including polymerization and aggregation—that affect the properties of the gluten network [7,8,9] and impact on protein solubility. The altered interaction among proteins in turn affects the solubility of suitable biomarkers in the solvent system used to prepare samples for subsequent immunochemical or analytical detection [3,10,11].

In most cases, preparation of samples for an immunochemical gluten detection procedure calls for solubilization of gluten proteins at high concentrations of aqueous ethanol or isopropanol (40% to 60%). The solubilization step is of paramount relevance. As an example of this point, several of the “gluten-removal” studies in the literature [9] did not verify whether a specific treatment had an impact on protein solubility in the solvent system used to prepare samples for subsequent analysis [3,10,11]. As a consequence, some of the claims about physical treatments assumingly capable of removing offending peptides have been challenged by subsequent studies [12].

An example of the effects of process-dependent protein modification on the analytical issues (recovery and immunochemical reactivity) mentioned above is provided by studies on the impact of a combination of high hydrostatic pressure (HHP) and thermal treatment on the solubility and immunoreactivity of gluten proteins. In these studies, an aqueous flour suspension (40% solids) was treated for 10 min at 600 MPa while kept at a set temperature in the 40–110 °C range. This combination of treatments (HHP and temperature) is expected to promote the formation of protein aggregates stabilized by both non-covalent hydrophobic interactions and disulfide bonds originating from intra- and intermolecular disulfide exchange reactions in a treatment-dependent fashion [13].

Proteins in dough from the HHP-treated samples were solubilized either with aqueous ethanol (60% *v*/*v*) or with a chaotrope/reductant mixture (6 M urea and 10 mM DTT in 20 mM phosphate buffer, pH 7.0). These two combinations represent common media used for the solubilization of cereal proteins [5,13]. The solubilized proteins were then used for the immunochemical detection of gliadin.

**Figure 1:**
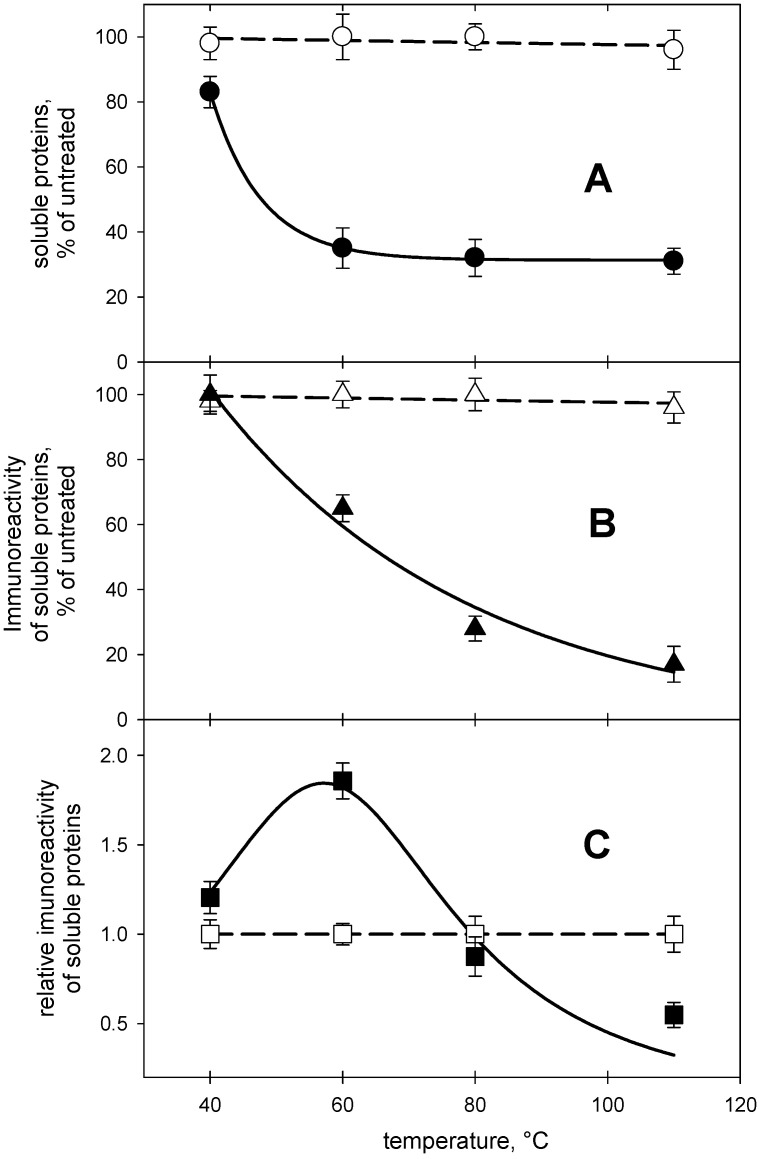
Effects of temperature during high hydrostatic pressure treatment (600 MPa, 10 min) of aqueous flour suspensions (40% solids). (**A**): protein solubility in 60% aqueous ethanol (full symbols) or 6M urea and 10 mM DTT (open symbols). (**B**): immunoreactivity of the proteins solubilized by individual extractants. (**C**): relative immunoreactivity of solubilized proteins. Symbols in the two upper panels are slightly larger than the standard deviation from quadruplicate measurements in duplicated experiments. Routines embedded in the graphical software SigmaPlot (rev. 10, Jandel Scientific, San Rafael, CA, USA) were used for data analysis and curve fitting.

The data in Figure 1A clearly indicate that the choice of the solubilizing agent affects the recovery of gluten proteins in a soluble form. The chaotrope/reductant combination appeared capable of solubilizing essentially the same amount of proteins that was solubilized from untreated flour suspensions regardless of the temperature at which the HHP treatment was carried out. As expected, the proteins solubilized by the urea/DTT mixture retained their ability to be recognized by the antibodies used in the “standard” R5-based assay that is based on recognition of a sequence-based epitope (Figure 1B).

On the contrary, the amount of proteins solubilized by aqueous ethanol decreased sensibly in HP-treated samples. Only 30% of the proteins were soluble in aqueous ethanol when the temperature during the HP treatment was kept above 60 °C (Figure 1A). As expected, decreased protein solubility in aqueous ethanol at increased treatment intensity was accompanied by a decrease in immunoreactivity of the extracted materials towards the R5 antibody (Figure 1B). However, the decrease in immunoreactivity of the proteins solubilized by aqueous ethanol at increasing treatment intensity cannot be simply explained by the protein solubility profile, as underscored by the data in Figure 1C, where the relative abundance of immunoreactive protein appears to reach a maximum at temperatures around 60 °C.

One possible explanation for the results in Figure 1 may come from the molecular characteristics of the involved proteins [14]. Gliadins (that bear the QQPFP sequence recognized by the R5 antibody) do not contain free thiols, as all of the cysteine residues in gliadins are involved in intramolecular disulfide bridges. These molecular features make it possible to use aqueous ethanol to solubilize gliadins from untreated flour, with only marginal effects of treatment temperature on the extraction yield. However, the HP treatment promotes structural rearrangements [13] that may generate species that undergo further structural rearrangements and novel chemical reactivity when temperature is increased during the HP treatment itself. In the case discussed here, these events—at temperatures below 60 °C—make immunoreactive gluten proteins reach the highest relative abundance in the material solubilized by aqueous ethanol from flour suspension that were HP-treated at 60 °C. When the temperature during the HP treatment is increased further, so does the possibility that the disulfide bonds in gliadins become involved in disulfide exchange reactions. The result is the formation of a disulfide-linked network of all immunoreactive proteins that is pretty much insoluble in aqueous ethanol and soluble only when chaotropes and thiols are present in the extraction medium to break down both hydrophobic interactions and disulfide bonds, respectively.

### 2.2. Detection of Non-Gluten Wheat Proteins

Foods containing whole grain cereals are assumed to provide a number of health benefits over those based on refined grains [15,16]. A standardized definition of whole grain foods could form the basis for dietary recommendations, for accurate labelling of whole grain products, and for providing consistent and clear information to consumers, to researchers in the field, and to the food industry at large [17]. Whole grain foods are difficult to be legally defined because validated methods are not available and because those that have been proposed are time consuming and expensive [18,19,20].

In this complex context, wheat germ agglutinin (WGA), a plant lectin common among *Triticum* species, [21] has been suggested as a possible biomarker of the presence of wheat germ in wheat-based products [22]. WGA is predominantly expressed in wheat germ, where it is component of the immune system of the plant [23].

A number of methods were set up for WGA purification from wheat germ [21,24], and analytical approaches based on protein purification have been proposed [25]. These methods may be regarded only as semi-quantitative, being strongly impaired by interference by other food components. Methods based on the lectin activity of the protein have also been reported [26,27,28,29]. Commercial antibodies towards WGA have been used for Western blot analysis of SDS-PAGE tracings of proteins solubilized by dilute hydrochloric acid from flour and pasta samples [30].

The possible alternative tested here uses a straightforward ELISA approach for quantification of WGA in extracts obtained from variously processed wheat-derived products by treatment with dilute solutions of acids or bases. Such a simplified procedure could overcome: (1) the likely solubility issues ensuing from extraction in buffered saline, in particular when treating processed foods; (2) the loss of some biological activities as a consequence of thermal processes [30]; (3) the inherent complexity and difficulties associated with quantitative analysis of Western blot measurements [30]; (4) the possible issues related to the poor specificity of commercially available anti-WGA antibodies [31], although commercial antibodies have been shown to recognize WGA even when WGA was denatured by heat treatment in the presence of detergents [30].

**Figure 2:**
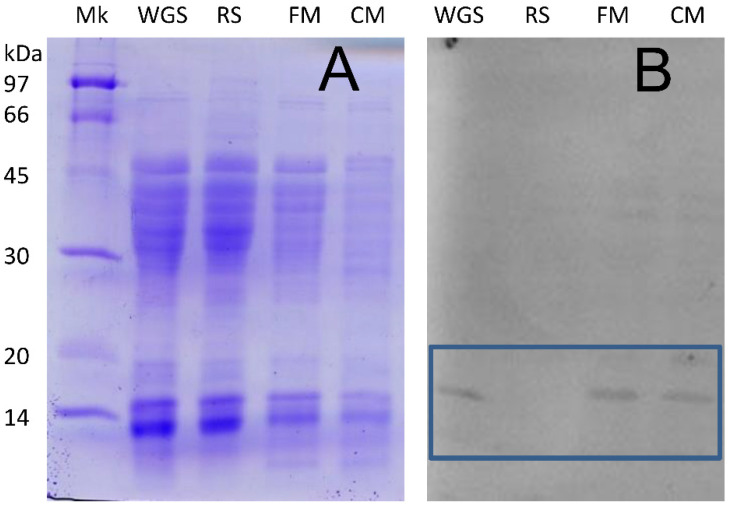
SDS-PAGE and Western blotting tracings for WGA extracts. The samples are identified as follows: markers (Mk); whole grain semolina (WGS); refined semolina (RS); fine millings (FM); coarse millings (CM). (**A**): Coomassie Blue staining; (**B**): Western blotting against commercial anti-WGA antibodies.

In this study, WGA was at first isolated from various matrices (whole grain semolina, fine and coarse milling fractions from durum wheat, and whole grain pasta) using extraction with HCl in the presence of a reducing agent and further purified by precipitation with 35% ammonium sulfate and ion-exchange chromatography. The SDS-PAGE tracings presented in Figure 2A indicate that that ion exchange chromatography on the neutralized acid extracts only provided a very coarse separation of proteins, so that this procedure does not seem suitable for protein identification or quantification.

Western blotting of the proteins present in the various extracts and separated by SDS-PAGE (Figure 2B) indicated the presence of the WGA only in whole grain semolina or in fractions containing bran, such as various milled bran fractions, but came of providing a reliable quantitation of the protein. The detection of WGA in both coarse and fine millings (CM and FM in Figure 2) highlights the difficulties associated with the complete removal of the germ during the milling of durum wheat.

To confirm the indications from Western blotting data, we analyzed the same samples previously analyzed by SDS-PAGE to assess the presence of WGA-derived peptides by LC-MS/MS after appropriate proteolytic treatments. This approach confirmed the presence of agglutinin isolectin 2 (WGA2) (accession number P02876) in the whole grain extracts (see Table S1) and the absence of this protein component from the refined semolina sample.

Following the detection of WGA in the extracts from whole grain milling fractions, an indirect competitive ELISA was set up for its quantification. According to the resulting standard curve (not shown), the protocol allowed detection of WGA in the 0.03–4 microgram range, which appears suitable for WGA quantification, as the WGA content in wheat germ reportedly ranges from 6 to 8 microgram/g of caryopsis [21].

The amount of WGA detected in the various extracts is reported in Table 1. At least 90% of the WGA expected to be present in the durum wheat kernel could be determined by competitive ELISA in the acid-extracted/enriched materials from whole grain semolina. A roughly ten-fold lower amount of WGA was detected in both fine and coarse millings with respect to whole grain semolina, confirming incomplete removal of the germ during industrial milling. From a methodological standpoint, it should be noted that the figures reported in Table 1 did not change more than 2% when testing various concentrations of DTT (from 0 to 20 mM) in the extraction steps, or when substituting equivalent concentrations of 2-mercaptoethanol for DTT. Although this indicates the absence of disulfide-linked WGA in these materials, it is worth remembering that both mono- and dithiols have modest disulfide-breaking capacity at low pH values.

However, when similar extraction and quantitation protocols were applied to pasta samples (including one made from the same whole grain semolina used for the analyses reported in Table 1), it was impossible to detect or quantitate WGA in any of the pasta extracts. Instead, WGA was detectable in pasta extracts obtained by replacing the diluted HCl called for by the original extraction procedure [21] with 50 mM NaOH. This substitution did not require major rearrangements in the subsequent enrichment steps other than neutralization of the initial extract. Both MS and Western blotting confirmed the presence of WGA in enriched alkaline pasta extracts (not shown).

These findings allowed a quantitative estimate of the amount of WGA detectable by ELISA in enriched alkaline extracts from two different samples of pasta. As reported in Table 2, results obtained from pasta made from the same whole grain semolina analyzed in Table 1 indicate a decrease in the WGA amount in pasta with respect to un-processed semolina (from 5.7 to about 2 microgram/gram).

This indicates that some of the steps involved in pasta processing lead to the formation of a protein network that may include or entrap WGA, preventing its release and quantification. By considering the abundance of cysteine residues in WGA (28 cysteine residues out of 165 amino acids), it seems likely that WGA may be involved in disulfide exchange events leading to the formation of inter-chain disulfide bonds with gluten proteins during gluten network formation in either the kneading or the drying steps of pasta-making. However, it has to be noted that the presence of 2-mercaptoethanol during the alkaline extraction of pasta—as routinely performed in this study—did not appear to help in releasing WGA from the very compact protein network in whole grain pasta. A decreased recovery of WGA from processed samples provides a more likely explanation for these results than a decreased WGA immunoreactivity. WGA was reportedly found to retain its immunoreactivity (but not its lectin-like activity) upon treatment at high temperatures or after being treated at very alkaline pH values [29].

Impaired WGA recovery from whole grain pasta samples was somehow expected since the structural modifications that accompany the mixing, forming, and—most notably—the drying steps in pasta making are reportedly affecting the compactness of the protein network [32,33,34,35], so that high-temperature drying may form protein aggregates that are way too compact to be dissociated by disulfide reductants under the conditions typically used in these studies [35,36]. The formation of large multi-protein polymers induced by elevated drying temperatures [32,33,34,35] may result in a decreased extractability of WGA as a consequence of increased integration or inclusion in the gluten network. As discussed in a previous case presented in this report, similar issues affect recovery of other thiol-rich proteins—such as gluten or egg white ovalbumin—from processed wheat-based foods [7,37], so that specific extraction procedures are required to ensure the accuracy and repeatability of protein quantitation in subsequent immunochemical analysis [38,39].

Table 2 also compares a commercial pasta made from the same whole grain semolina characterized in Table 1 with another commercial pasta labeled as “wheat germ containing”. We had no access to the raw materials used for this particular production. Assuming that both pasta samples were made from whole grain semolina having similar WGA content, the data in Table 2 suggest that the intensity/severity of pasta drying conditions played a major role in WGA extractability. As indicated by their furosine content, the pasta samples used for WGA determination in Table 2 underwent drying cycles of different severity. Increased severity of the drying process conditions (resulting in an increased furosine content) relates to impaired WGA detection, being ten times lower in pasta samples dried at high temperature than in pasta dried at low temperature.

Thus, the data reported here indicate that detection of WGA in whole grain pasta was strongly influenced by the mixing and drying steps in the industrial pasta production process. In addition, less than 50% of the WGA present in whole grain semolina was found already in pasta prepared by using a low-temperature drying cycle, pointing to effects of shear forces on gluten proteins and on the ensuing rearrangement of their intermolecular bonds during mixing/forming.

### 2.3. Detection of Non-Wheat Proteins in Pasta

Quantification of food allergens represents an important issue for the food industry, being fundamental to ensure consumer safety. Eggs are a potential source of protein allergens as well as of proteins with anti-nutritional properties. In some cases, as it is for ovalbumin (OVA), these negative traits are related to the same molecule, as OVA is both a well-known allergen and a protease inhibitor in its native form.

In common practice, quantitation of egg-derived allergens most often relies on immunochemical detection of ovalbumin, a glycated protein that has an unusually high immunoreactivity. In spite of the abundance of ovalbumin in egg white and of the remarkable sensitivity of the available immunochemical assays [40], reliability and ease of use of commercial kits for detection of ovalbumin can be challenging, either because ovalbumin is very prone to forming macropolymers as a consequence of processing or of noticeable changes in immunoreactivity when ovalbumin or egg white undergo processes that may modify protein structure and reactivity in ways that are also affected by the presence of other common food ingredients, such as sugars or salt [41,42].

Treatment-related protein polymerization of albumin is of particular relevance in cereal-based foods, as egg proteins may be incorporated into or entrapped within the gluten network [7]. Egg proteins may end up as being part of the resulting protein network itself, or may be present as polymers way too large to escape the cage-like network formed by proteins of plant origin, even in the case of gluten-free formulations [7,8]. Simultaneous or independent occurrence of inclusion/entrapment events makes the solubilization of egg proteins very difficult, if not impossible, even in cases where physical treatments are limited in their intensity [38].

The scenario discussed above is further complicated when dealing with low concentrations of egg proteins, as happens in the pasta industry when shifting production lines from egg-containing to semolina-only pasta. This procedure is commonplace in the pasta industry and calls for careful and timely control of the production lines to make semolina-only pasta safe for the sensitive consumer, while minimizing the waste of time and materials and keeping costs reasonable, as analyzed in some recent reports [39].

Table 3 presents the time course of the decrease in the amount of egg proteins detected in pasta coming out from an industrial plant (>2000 kg/h) immediately after switching from the production of egg-containing pasta to that of semolina-only pasta. In this study, egg-proteins were detected by using two different immunochemical kits, both commercially available, that are based on the same antibody and only differ as for the composition of the buffer used for protein solubilization [40]. The data presented in Table 3 clearly indicate that protein solubilization—and therefore their detection—was strongly influenced by the composition of the buffer used in preparation of the samples. The presence of the disulfide reductant 2-mercaptoethanol (2-ME) in the “M” extraction buffer greatly improved recovery of OVA from fresh pasta samples (roughly twice the amount extracted by the buffer in the “R” kit). The difference between the two types of extractant is even more dramatic in the case of dried pasta, where only about 25% of the egg proteins were solubilized in the absence of a disulfide reductant.

From a molecular standpoint, the observed differences in protein solubility may be taken as an indication that a disulfide-linked network is already formed by egg proteins during the kneading step of egg-containing pasta. The resulting network is further stabilized by structural modifications occurring during the drying process, which further stabilize the inter-protein network. In this frame, it has to be noted that the protein extraction procedure suggested for the “R” kit involves incubation at 60 °C for 10 min, in contrast with the overnight incubation at room temperature suggested for the “M” kit. Nevertheless, the detergent/temperature combined treatment seems inadequate for solubilization of egg proteins, indicating a strict requirement for inclusion of a reductant in the extraction buffer and suggesting a significant role of disulfide bonds in the stabilization of the protein network in the final product.

The practical implications of these results may be considered as self-evident, as using improper solubilization procedures may lead to considering as safe (at least from the standpoint of egg proteins content) products that still contain a significant amount of ovalbumin. This is exemplified by the results obtained on dried pasta 20 min after shifting the production line from egg-containing to semolina-only pasta (Table 3). Given that—under the strictest legislation in the Authors’ own country—residual allergens must be “below the detection level of the most sensitive assay” [43], it appears clear that the plant used in this study must be operated for at least 60 min after switching production lines (i.e., must produce 2000 kg of pasta, in the industrial plant used in this study) before the product could be considered egg-free, even without taking into consideration other relevant factors, such as the shape and size of the final product [39].

## 3. Materials and Methods

### 3.1. Materials

In all cases, chemicals were of reagent grade or better and were from Sigma-Aldrich (Milan, Italy) unless otherwise indicated. Commercial refined common wheat flour was used for HHP/temperature treatments, carried out in a Pressure Flow HHP apparatus described in previous reports [41,42]. Deionized water was added to a proper amount (50 g) of flour to reach 40% solids (*w*/*v*) under manual stirring. The resulting slurry was placed in a sealed plastic bag that was placed in the high pressure chamber filled with water at appropriate temperature. Pressure treatment was carried out at 600 MPa for 10 min, while monitoring temperature inside the vessel through the apparatus’ own sensors. Immediately after the pressure treatment, the treated samples were removed from the vessel, refrigerated in an ice/water bath, and kept overnight at 4 °C prior to further analysis.

Pasta samples for ovalbumin detection studies were provided by Valdigrano SpA, Rovato, Italy, and were produced in an industrial line having a production capacity of 2.3 tons/hour. Pasta samples were taken at various times after shifting the feed of the production line from egg-containing to semolina-only and after having cleaned the production line from residual materials. Pasta samples were taken immediately after the extrusion step (“fresh pasta”) and at the end of the subsequent drying step (“dried pasta”). Samples of fresh pasta were frozen at −80 °C and lyophilized (Alpha 2–4 LD, Christ, Osterhode am Harz, Germany) prior to further characterization. Both fresh and dried pasta samples were ground to <250 µm in a laboratory mill prior to analytical determinations.

Smashed grains, refined semolina, whole-grain semolina, fine and coarse millings from durum wheat (*Triticum durum*) used in studies on WGA were provided by F.lli De Cecco SpA (Fara S. Martino, Italy), that also supplied a whole grain pasta produced in their industrial plant from the same whole-grain semolina. Whole-grain pasta from another Italian producer was purchased locally.

### 3.2. Protein Solubilization, Enrichment, and Identification

In the case of HPP/temperature treatments, solids in individual treated and untreated slurries were recovered by low-speed centrifugation, washed twice with water, and suspended either in 60% (*v*/*v*) aqueous ethanol or in 50 mM phosphate buffer, pH 7.0, containing 6 M urea and 10 mM dithiothreitol (DTT). After 1 h stirring of each suspension at room temperature, solutions for subsequent immunochemical analysis were recovered by high-speed centrifugation (12,000× *g* for 15 min at 4 °C), appropriately diluted, and used immediately.

For enrichment purposes, WGA extraction from the various milling products and from pasta samples was carried out by minor modifications of a standard acid-extraction procedure [21]. However, the acid-extraction protocol was found to be ineffective in WGA recovery from pasta samples, which therefore were ground to a 0.3 mm particle size and treated with 0.05 M NaOH in the presence of 10 mM DTT for 2 h at 4 °C, followed by neutralization and by further overnight stirring. Both neutralized suspensions were centrifuged at 8000× *g* for 90 min at 15 °C. For further purification, proteins were precipitated with ammonium sulfate (35% saturation, 1 h at 4 °C), dialyzed against 0.05 M Tris-HCl buffer, pH 7.7, and loaded onto a DEAE–Sepharose^®^ Fast Flow (Sigma-Aldrich, Milan, Italy) column, equilibrated in 0.05 M Tris-HCl buffer, pH 7.7. WGA in the unbound material was recovered and concentrated on a 3 kDa ultrafiltration membrane. The concentrated extracts were analyzed by SDS-PAGE under reducing conditions, essentially as reported previously [5]. Briefly, protein samples were diluted with denaturing buffer (0.125 M Tris-HCl, pH 6.8; 50% glycerol (*v*/*v*); 17 g/L SDS; 0.1 g/L Bromophenol Blue) containing 10 mL/L of 2-mercaptoethanol and heated at 100 °C for 10 min. SDS-PAGE was carried out in a MiniProtein apparatus (Bio-Rad, Richmond, VA, USA) using a Tris/glycine buffer system. Gels were stained with Coomassie Blue.

For liquid chromatography tandem mass spectrometry (LC-MS/MS), WGA-containing neutralized extracts were reduced with 5 mM DTT and alkylated with iodoacetamide according to standard proteomics protocols. Samples were desalted against 0.025 M ammonium bicarbonate, pH 7.2, and incubated overnight at 37 °C with 0.015 mg/mL proteomic grade trypsin (Promega, Madison, WI, USA). The resulting peptides were desalted on Sep-Pak^®^ C18 cartridges (Waters, Milford, MA, USA) and separated using an Ultimate 3000 system (Dionex/Thermo Fisher Scientific, San Jose, CA, USA), coupled to a Q-Exactive Orbitrap mass spectrometer (Thermo Fisher Scientific). Peptide mixtures in 0.1% (*v*/*v*) formic acid (FA) were loaded through a 5 mm long, 300 μm i.d. pre-column (LC Packings, USA), and separated with an EASY-Spray™ PepMap C18 column (3 μm particles, 100 Å pore size, 15 cm × 75 μm (Thermo Fisher Scientific)) operating at 300 nL/min flow of 0.1% FA (*v*/*v*) in Milli-Q water with a linear gradient from 2% to 50% 0.1% FA (*v*/*v*) in acetonitrile over 60 min. Mass spectra were generated by the Xcalibur Software (rev. 3.1) and elaborated against the Uniprot database (http://prospector.ucsf.edu/prospector/mshome.htm, accessed on 12 November 2021). Entries were filtered by using: *Triticum* taxonomy restriction, trypsin enzyme, 20 ppm tolerance for precursor ions and 0.08 Da for MS/MS fragments, oxidized Met and N-terminus Gln as pyroglutamic acid as variable peptide modifications, and by restricting the search at 0.01 false discovery rate (FDR).

### 3.3. Immunochemistry

ELISA assays for gluten were performed by using commercial kits, as recommended by the producer (R-Biopharm) and reported elsewhere [13]. Commercially available kits (“Egg proteins”, from R-Biopharm, “R”; “Egg white”, from Morinaga, “M”) were used for ovalbumin detection following recommendations by the supplier. In the case of WGA, an ELISA calibration curve was constructed using commercial purified WGA (Sigma-Aldrich, Milan, Italy, 16 to 2000 ng/well) and an anti-WGA rabbit polyclonal antibody (dilution 1:40,000; Sigma-Aldrich, Milan, Italy). For analytical purposes, various dilutions of the supernatants from the extraction procedure were incubated with the antibody for 1 h prior to transfer into wells previously coated with 0.5 µg/well of WGA in 50 mM sodium carbonate buffer, pH 9.5. After 1 h, plates were thoroughly washed with PBS–Tween buffer, followed by addition of an anti-rabbit IgG-horseradish peroxidase conjugate (0.1 mL/well, dilution 1:3000; Sigma-Aldrich, Milan, Italy), incubation for 45 min, and washing with PBS–Tween buffer. Color development used 0.1 mL/well of 6 mM 3,3′,5,5′-tetramethylbenzidine in peroxide buffer (40 mM sodium acetate, 3.2 mM sodium perborate, pH 5.0), and was stopped after 10 min by adding 0.1 mL/well of 2 M H_3_PO_4_ prior to analysis in an automated microplate reader (Bio-Rad, Milan, Italy).

For Western blotting, proteins in the gel were transferred to nitrocellulose membranes that were then blocked with 0.25% whey proteins, thoroughly washed with 0.05 M Tris-HCl, pH 7.6, containing 0.15 M NaCl and 0.05% Tween-20 (*v*/*v*), and incubated for 2 h with a rabbit anti-WGA polyclonal antibody (Abcam, Cambridge, UK) diluted 1000-fold in the same buffer used for the washing steps. WGA-bound antibodies were detected by using an anti-rabbit IgG-peroxidase conjugate and SIGMAFAST™ DAB tablets (both from Sigma-Aldrich, Milan, Italy).

### 3.4. Data Analysis

All the data presented in Table 1, Table 2 and Table 3 are the average of three replicates from two independent measurements. The data in the two upper panels of Figure 1 are the average of quadruplicate determinations in two independent experiments. Routines embedded in the graphical software SigmaPlot (rev. 10, Jandel Scientific, San Rafael, CA, USA) were used for data analysis and curve fitting in Figure 1.

## 4. Conclusions

The molecular basis of the observed behavior in individual process/protein cases appear to involve distinctive traits in individual cases. In general, the inverse relationship between treatment intensity and analytical recovery has been shown to be far from linear, as a consequence of the complex steps involved in the formation of non-extractable or otherwise undetectable species. This offers a first cautionary message as for generalizing observations made on a given food product/process to systems that appear to be similar either in their components or in terms of the treatments/processes they may undergo. The relative amount of involved proteins, their sensitivity to unfolding and the individual kinetics of unfolding for a specific protein, and the time course of events that lead to interaction with other macromolecules all play a role in these events. Whereas these points have been addressed quite extensively in the case of soluble food proteins [41,42,44,45], systems that include intrinsically unfolded or insoluble proteins seem worth further and detailed investigation, so that the practical aspects mentioned for each of the three cases presented here above could be assessed on a more solid and circumstantiated molecular basis.

In more detail, in the wheat-based food systems considered here, intrinsically unfolded proteins such as gliadins interact with proteins in a system (such as the intrinsically unfolded glutenins) through both covalent and non-covalent interactions. Suitably reactive conformers capable of establishing hydrophobic interaction may, for instance, appear at a given treatment intensity but be no longer present above a certain intensity/time threshold. Needless to say, the same reasoning applies to the formation of individual protein conformers prone to undergo disulfide exchange reactions. This provides a coarse and highly simplified background for explaining the complicated dependence of analyte recovery on the treatment parameters, as exemplified in Figure 1, where the additional complication of simultaneous application of different physical unfolding agents comes into play—a common occurrence in food processing.

Adding folded proteins (WGA or OVA) to the scene will hardly improve legibility of such a complicated plot, in particular when considering that WGA contains 32 cysteine residues (all involved in 16 intramolecular disulfide bonds), whereas OVA has 6 cysteine residues, of which only 2 are forming an intramolecular disulfide. Moreover, because of this, the two proteins differ in their sensitivity to unfolding agents, as OVA is much more sensitive to denaturants than WGA). The different thiol/disulfide pattern also provides a mechanistic rationale for a remarkably different behavior in the case of OVA or WGA that may be engaging in intermolecular disulfide exchange with gluten proteins, as in the cases presented here. From a practical standpoint, structural differences in individual biomarker proteins should warn against generalizing the outcome of individual studies.

Thus, conditions need to be found—on a case-by-case basis—whenever the recovery of a given protein biomarker needs to be improved and optimized. In the relatively simple cases discussed here, use of appropriate dissociating agents (such as chaotropes and thiols rather than aqueous ethanol or isopropanol for gluten solubilization, or the use of alkaline extraction in place of an acidic one for WGA extraction from whole grain pasta) has been proven helpful. Admittedly, the situation can be expected to be more complicated when dealing with complex or involved food formulations, such as high-fat formulations and formulations with high levels of components of non-biological origin. For the sake of simplicity, none of these cases were considered in this particular report. Yet another source of trouble is the use of severe processing conditions, as exemplified here by the sensitivity of WGA recovery from whole grain pasta to the conditions used in the drying steps (see Table 3).

In conclusion, case studies such as the ones presented here highlight some points that appear to be of practical relevance—namely: (1) physical treatments may affect recovery of a protein analyte in a way that does not depend in linear fashion from the treatment intensity, in particular when multiple interactions are simultaneously at play; (2) improvements need to be designed taking into due account the specific situation in terms of ingredients and their ratios, as well as the nature and intensity of the treatments used in concomitant/subsequent processing steps; (3) proper selection of the analytical procedure may help greatly in preventing coarse errors in the assay, false overestimation of the efficacy of a given treatment, inconsistent ability to manage safety and/or quality issues, subsequent troubles with consumers, and costly legal actions that may ensue from improper analytical practices.

## Figures and Tables

**Table 1:** Immunochemical detection of wheat germ agglutinin (WGA) in extracts from various semolina samples.

Sample	WGA (μg/g)
whole grain semolina	5.70 ± 0.38
refined semolina	<0.025
fine millings	0.50 ± 0.06
coarse millings	0.69 ± 0.09

**Table 2:** Wheat germ agglutinin (WGA) and furosine content in commercial whole grain pasta.

Sample	WGA (μg/g)	Furosine (mg/g Proteins)
Pasta 1 *	2.04 ± 0.14	3.50 ± 0.006
Pasta 2 ^§^	0.21 ± 0.03	8.36 ± 0.022

* Pasta 1 was made from the same whole grain semolina characterized in Table 1. ^§^ Pasta 2 was a commercial sample from a different producer, labeled as “contains wheat germ”.

**Table 3:** Time course of the decrease in egg protein content in pasta samples after the production switch from egg-based to semolina only pasta.

Time after Production Switch, min	Egg Protein Content (mg/kg)			
	Fresh Pasta		Dried Pasta	
	“R” Buffer + Detergent	“M” Buffer + Detergent + 2-ME	“R” Buffer + Detergent	“M” Buffer + Detergent + 2-ME
0	6.0 ± 0.7	13.7 ± 0.8	2.6 ± 0.2	11.1 ± 0.4
20	0.5 ± 0.04	1.4 ± 0.1	<0.25	1.1 ± 0.1
45	0.4 ± 0.04	0.6 ± 0.06	<0.25	<0.3
60	<0.25	<0.3	<0.25	<0.3
90	<0.25	<0.3	<0.25	<0.3

## Supplementary Materials

The following supporting information can be downloaded at: https://www.mdpi.com/article/10.3390/molecules27092637/s1, Table S1: Proteins identified by LC/MS in acid-extracted fractions from various durum wheat milling products. Uncharacterized proteins were removed from each list.
