# Supplementary material for: Quantification of Protein “Biomarkers” in Wheat-Based Food Systems: Dealing with Process-Related Issues
Source: Molecules. 2022 Apr 20;27(9):2637. doi: 10.3390/molecules27092637 (PMC9100356; doi:10.3390/molecules27092637)
Supplement: Supplementary file 1 [file molecules-27-02637-s001.zip › molecules-1643881-SI.pdf]

Table S1: Proteins id

WHOLE GRAIN SEMOLINA							
Rank	Acc #	Num Unique	% Cov	Best Disc Score	Best Expect Val	Protein MW	Protein pI
[1]	A4GFN8	10	90.3	10.30	1.8e-18	13181.4	6.5
[3]	P16347	11	73.9	6.86	4.3e-12	19633.2	6.8
[4]	Q0Q5E3	11	39.7	9.47	6.2e-17	24999.2	8.7
[5]	P17314	9	75.6	8.38	6.4e-15	18221.5	7.4
[7]	A4ZIT6	10	80.2	8.61	2.4e-15	13097.3	7.4
[8]	O64392	5	54.8	8.74	1.4e-15	15634.7	7.6
[9]	P16159	6	41.3	8.29	9.5e-15	15782.5	5.3
[11]	D2TGC2	5	72.5	10.49	8.0e-19	13004.9	5.8
[13]	D2KFH1	4	30.2	7.93	4.4e-14	18916.0	8.2
[14]	S4UM47	4	39.6	8.02	3.1e-14	15138.5	7.6
[15]	A7XDG3	4	45.5	6.95	3.0e-12	16205.8	8.9
[17]	D2KFH2	5	50.0	6.16	8.4e-11	11325.1	8.0
[18]	C3VWD9	4	32.6	8.88	7.8e-16	15024.4	4.8
[20]	D2KFH0	4	31.1	7.77	8.6e-14	20606.8	6.8
[21]	M8AQI5	3	11.8	14.07	1.9e-25	31758.8	8.5
[22]	Q5BHS7	3	23.8	8.78	1.2e-15	18142.9	5.5
[25]	Q41579	3	14.7	6.00	1.7e-10	23229.3	9.2
[28]	P24296	3	25.7	7.33	1.4e-13	11898.9	8.5
**[29]**	**P02876**	**3**	**10.3**	**5.55**	**2.9e-10**	**21356.4**	**7.8**
[32]	Q4Z8L8	3	23.3	5.79	9.1e-11	28218.0	8.7
[33]	B2Y2R3	2	8.9	6.66	1.0e-11	44641.0	8.9
[34]	W5B1E5	3	31.6	6.65	1.1e-11	15121.0	5.7
[35]	Q6UJY5	3	5.0	5.73	5.2e-10	88504.6	8.3
[36]	P82900	2	20.8	6.45	1.4e-11	9831.7	8.9
[37]	D3UAL6	4	9.5	3.02	5.9e-6	36283.3	8.2
[38]	P08488	3	4.7	4.28	1.8e-7	70867.8	7.6
[41]	Q9FEQ2	2	7.2	5.11	7.5e-9	44567.1	9.2
[42]	B7U6L5	2	6.5	5.26	3.8e-9	56931.5	7.4
[44]	Q4W1F9	2	26.6	5.45	6.3e-10	10439.3	8.4
[45]	M7Z628	3	20.1	3.53	7.1e-7	24035.7	6.1
[46]	C4P5B7	1	9.9	8.23	1.3e-14	16546.4	5.8
[47]	P42755	2	24.7	4.99	5.5e-9	10060.0	5.1
[48]	W5FJV5	2	9.0	4.51	9.6e-8	16539.2	11.0
[50]	B6UKS0	2	10.0	6.19	1.2e-11	34439.8	8.5
[51]	Q94G94	1	6.7	7.69	1.2e-13	32552.6	8.6
[52]	A0A1G4P206	1	4.0	7.68	1.3e-13	40111.4	8.9
[53]	P04726	3	13.5	3.23	6.0e-6	33941.6	8.8
[55]	P84971	1	33.3	7.25	5.0e-13	4989.8	8.5
[56]	Q84UH6	2	12.7	4.44	7.8e-8	23357.9	5.9
[58]	Q93W25	1	8.2	7.15	1.2e-12	18391.3	8.5
[59]	A4GFR0	1	20.2	6.87	4.1e-12	13301.4	5.3
[62]	Q93XQ6	1	8.2	6.56	1.5e-11	18379.2	8.5
[65]	P30569	1	17.3	6.32	4.5e-13	7712.7	7.9
[67]	I0IT53	1	5.1	5.52	4.0e-10	33611.1	7.6
[69]	Q9FEQ1	1	5.0	5.27	4.4e-10	33880.6	7.6
[70]	P0CZ05	2	7.7	3.07	3.2e-7	32513.8	7.8
[72]	Q75QN8	1	8.7	5.09	4.9e-10	21543.9	5.7

[73]	R9XV30	1	4.2	4.95	1.2e-9	32920.5	8.3
[77]	W5AY74	1	6.6	4.35	2.8e-8	27692.6	6.3
[79]	W5AQ78	1	4.0	4.14	1.5e-8	34252.9	6.1
[83]	O22116	1	3.5	3.94	1.1e-6	43114.4	8.9
[84]	Q43665	1	13.5	3.91	1.6e-9	9518.3	8.4
[85]	R4VEK6	1	5.3	3.87	1.4e-7	32566.3	8.6
[87]	B1PDK7	1	3.7	3.81	1.9e-6	33941.8	8.6
[88]	I6U6I8	1	3.2	3.78	2.2e-6	35920.5	6.6
[91]	A0A0M4FCD1	1	8.8	3.54	7.9e-8	12603.4	5.7
[94]	W5D0E3	1	3.4	3.30	8.5e-8	38895.8	6.8
[95]	K7X0W1	1	3.0	3.25	7.9e-7	33811.9	7.6
[96]	M7YAM8	1	10.5	3.17	2.7e-7	18276.3	8.7
[97]	R9XV62	1	7.6	3.16	6.2e-7	34462.5	6.9
[98]	R9XWD0	1	4.6	3.08	9.2e-8	37229.8	8.7
[99]	A2IBV5	1	2.8	3.01	3.3e-7	40435.1	8.9
[100]	A0A060AFB6	1	14.8	2.94	8.3e-7	8731.4	9.0
[103]	C3VWI4	1	6.4	2.77	2.1e-7	15308.7	6.1
[109]	M7ZZV2	2	4.3	1.70	4.5e-5	23557.9	7.8
[110]	P01544	2	12.7	1.28	2.4e-5	13525.7	4.8
[111]	Q7M219	1	44.0	2.20	8.7e-7	2764.2	4.9
[112]	Q07810	1	3.6	2.05	2.9e-6	29613.1	9.7
[114]	P81713	1	14.1	1.99	3.0e-6	7962.5	8.1
[115]	Q8LK23	1	3.4	1.96	5.1e-6	38823.5	8.1
[117]	Q2MCJ8	1	1.6	1.80	2.2e-4	65917.5	6.9
[118]	U5HTD8	1	9.4	1.74	9.1e-6	14105.6	9.5
[119]	Q7X9L9	1	6.7	1.60	7.3e-5	20090.9	10.5
[120]	M7ZW67	1	4.5	1.45	5.8e-5	17280.8	6.2
[121]	R9XUS2	1	6.7	1.39	6.4e-5	32663.6	8.8
[122]	A7LHB4	1	4.2	1.36	3.5e-5	33014.7	7.7
[123]	A0A1D5XQU5	1	3.6	1.28	6.3e-5	25092.4	10.1
[124]	P93790	1	3.4	1.27	5.2e-5	33906.0	9.0
[125]	R4ZA22	1	2.3	1.21	1.9e-4	39817.9	5.9
[126]	B8YM21	2	11.8	0.87	5.2e-5	14644.1	4.8
[127]	A0A1D5VJ57	1	2.8	0.85	0.0016	61220.5	7.9
[129]	Q43660	1	4.5	0.63	8.1e-4	17353.4	5.6
[131]	M7YZC5	1	3.4	0.56	0.0012	36745.1	9.4
[132]	P83207	1	8.4	0.54	0.0034	12944.1	7.4
[133]	P22701	1	9.6	0.41	0.0022	9986.9	5.3
[134]	A0A1D5V021	1	5.8	0.35	0.0045	13126.2	7.8

dentifed by LC/MS in acid-extracted fractions from various durum wheat milling products. Uncharacterized proteins we

Species	Protein Name	Rank	Acc #	Num Unique	% Cov
TRIDC	Dimeric alpha-amylase inhibitor (Fragment)	[1]	A4GFN8	9	90.3
WHEAT	Endogenous alpha-amylase/subtilisin inhibitor	[2]	A0A1S6KXP9	7	75.9
WHEAT	Globulin 1	[4]	A4ZIT6	6	52.9
WHEAT	Alpha-amylase/trypsin inhibitor CM3	[5]	D2TGC2	4	52.5
TRIMO	Monomeric alpha-amylase inhibitor (Fragment)	[6]	C3VWD9	2	24.1
WHEAT	Wheatwin-1	[8]	P16159	4	30.8
WHEAT	Alpha-amylase/trypsin inhibitor CM16	[10]	S4UM47	2	19.4
WHEAT	Putative alpha-amylase inhibitor CM2 (Fragment)	[13]	Q0Q5D9	2	10.2
WHEAT	Avenin-like a4	[14]	J7I1W7	1	6.2
TRIDC	Dimeric alpha-amylase inhibitor (Fragment)	[15]	M7ZZV2	2	4.3
WHEAT	Gamma gliadin	[16]	O49958	2	2.9
WHEAT	Gliadin/avenin-like seed protein (Fragment)	[17]	K7X0W1	2	3.0
TRIDC	Dimeric alpha-amylase inhibitor	[20]	Q9FEQ2	1	3.6
WHEAT	Gliadin/avenin-like seed protein	[21]	Q18MZ6	1	2.1
TRIUA	Avenin-3	[22]	P0CZ10	1	7.2
TRITU	GSP-1 Grain Softness Protein	[23]	A2IBV5	2	2.8
WHEAT	Rab protein	[24]	P0CZ05	2	7.7
WHEAT	Non-specific lipid-transfer protein (Fragment)	[25]	A7XDG3	1	9.7
**WHEAT**	**Agglutinin isolectin 2**	[26]	C3VWI4	1	6.4
WHEAT	Class II chitinase	[27]	W5B1E5	1	8.6
WHEAT	Low molecular weight glutenin subunit	[28]	Q43659	1	6.7
WHEAT	Superoxide dismutase [Cu-Zn]	**[30]**	**P02876**	**1**	**5.2**
TRITU	HMW-glutenin Bx subunit	[31]	P82900	1	9.4
WHEAT	Non-specific lipid-transfer protein 2G	[32]	M8AQI5	1	3.6
WHEAT	Low molecular weight glutenin subunit B3-1 (Fragment)	[33]	C4P598	1	6.6
WHEAT	Glutenin, high molecular weight subunit 12	[35]	B1PDK7	1	3.7
TRITD	Low molecular weight glutenin subunit (Fragment)	[36]	P24296	1	9.7
WHEAT	Globulin 3B	[37]	P93790	1	3.4
WHEAT	5a2 protein (Fragment)	[38]	D2KFH0	1	3.8
TRIUA	1-Cys peroxiredoxin PER1	[39]	O64394	1	8.7
TRIDC	Monomeric alpha-amylase inhibitor	[40]	I6U6I8	1	3.2
WHEAT	Em protein H5	[41]	R4ZA22	1	2.3
WHEAT	40S ribosomal protein S26	[45]	A0A0K0VHI4	1	3.3
TRIUA	Gamma-gliadin	[46]	C4P652	1	6.0
WHEAT	Gamma-gliadin				
WHEAT	HMW glutenin i-type subunit 3A				
WHEAT	Alpha/beta-gliadin clone PW1215				
TRIKH	Defensin Tk-AMP-D4				
WHEAT	Dehydroascorbate reductase				
WHEAT	Peptidyl-prolyl cis-trans isomerase				
TRIDC	Dimeric alpha-amylase inhibitor (Fragment)				
WHEAT	Peptidyl-prolyl cis-trans isomerase				
WHEAT	EC protein I/II				
WHEAT	Alpha/beta-gliadin				
TRITD	Low molecular weight glutenin subunit (Fragment)				
WHEAT	Avenin-like b2				
WHEAT	Cold shock domain protein 3				

WHEAT	Alpha-gliadin
WHEAT	Caleosin
WHEAT	Caleosin
WHEAT	LMM glutenin 3 (Fragment)
WHEAT	Wali5 protein
WHEAT	Gliadin
TRITU	Alpha-gliadin
TRIDC	Alpha-gliadin protein
TRIMO	Putative gliadin/avenin-like seed protein (Fragment)
WHEAT	Fructose-bisphosphate aldolase
WHEAT	Low molecular weight glutenin subunit
TRIUA	40S ribosomal protein S27
WHEAT	Gamma-gliadin
WHEAT	Gamma-gliadin
WHEAT	Glutenin subunit
WHEAT	Defensin
TRIDC	Dimeric alpha-amylase inhibitor
TRIUA	Alpha/beta-gliadin MM1
WHEAT	Alpha-1-purothionin (Fragment)
TRITD	Alpha-amylase inhibitor (Fragment)
WHEAT	rRNA N-glycosidase
WHEAT	Bowman-Birk type trypsin inhibitor
WHEAT	Peroxidase
TRIUA	High molecular weight glutenin subunit
WHEAT	Ribosomal protein S20
WHEAT	QM (Fragment)
TRIUA	Alpha-amylase/trypsin inhibitor CM2
WHEAT	Gamma-gliadin
WHEAT	Alpha gliadin
WHEAT	Oleosin
WHEAT	Low-molecular-weight glutenin storage protein
TRIKH	L-1
TRIUA	Beta purothionin
WHEAT	E3 ubiquitin-protein ligase
WHEAT	Heat shock protein 17.3
TRIUA	Serine/threonine-protein kinase CTR1
WHEAT	Chymotrypsin inhibitor WCI
WHEAT	Em protein CS41
WHEAT	Peroxiredoxin

ere removed from each list.

FINE MIDDLINGS

Best Disc Score	Best Expect Val	Protein MW	Protein pI	Species
9.77	1.7e-17	13181.4	6.5	TRIDC
7.28	7.0e-13	14926.4	7.7	WHEAT
7.88	5.6e-14	13097.3	7.4	TRIMO
8.71	1.6e-15	13004.9	5.8	WHEAT
9.26	1.5e-16	15024.4	4.8	TRIDC
5.25	4.0e-9	15782.5	5.3	WHEAT
6.64	1.1e-11	15138.5	7.6	TRIDC
3.66	2.2e-6	24550.7	8.6	WHEAT
6.55	1.6e-11	29503.0	8.7	TRIMO
3.59	3.1e-6	23557.9	7.8	TRIUA
3.25	9.8e-7	39791.5	8.1	TRITD
3.44	1.0e-6	33811.9	7.6	WHEAT
4.77	3.1e-8	44567.1	9.2	TRITD
4.60	2.9e-8	80069.8	8.8	WHEAT
4.43	2.6e-9	19798.0	8.2	WHEAT
2.03	1.4e-4	40435.1	8.9	WHEAT
2.13	1.5e-5	32513.8	7.8	WHEAT
3.73	5.8e-9	16205.8	8.9	WHEAT
3.62	2.4e-8	15308.7	6.1	TRIDC
3.56	7.6e-7	15121.0	5.7	WHEAT
3.37	1.7e-8	18449.5	8.0	WHEAT
**3.17**	**2.1e-6**	**21356.4**	**7.8**	**WHEAT**
2.86	3.9e-7	9831.7	8.9	WHEAT
2.76	1.4e-5	31758.8	8.5	TRIUA
2.31	1.2e-6	16513.4	7.4	TRIDC
2.21	1.9e-5	33941.8	8.6	TRITU
2.14	3.0e-5	11898.9	8.5	WHEAT
2.13	1.3e-4	33906.0	9.0	WHEAT
1.94	6.0e-6	20606.8	6.8	WHEAT
1.89	2.6e-5	13523.9	5.1	WHEAT
1.68	1.9e-5	35920.5	6.6	TRIDC
1.63	2.6e-4	39817.9	5.9	TRIKH
1.13	3.6e-4	31402.3	7.8	WHEAT
0.25	0.0050	16601.5	6.5	TRIDC

Protein Name
Dimeric alpha-amylase inhibitor (Fragment)
Alpha amylase/trypsin inhibitor (Fragment)
Monomeric alpha-amylase inhibitor (Fragment)
Putative alpha-amylase inhibitor CM2 (Fragment)
Dimeric alpha-amylase inhibitor
Alpha-amylase/trypsin inhibitor CM16
Dimeric alpha-amylase inhibitor (Fragment)
Globulin 1
Gamma-gliadin (Fragment)
Alpha/beta-gliadin MM1
Low molecular weight glutenin subunit (Fragment)
Low molecular weight glutenin subunit
Low molecular weight glutenin subunit (Fragment)
High-molecular-weight glutenin subunit Bx17
Avenin-like a6
Glutenin subunit
Avenin-like b2
Gamma gliadin
Dimeric alpha-amylase inhibitor
Superoxide dismutase [Cu-Zn]
15kDa grain softness protein
**Agglutinin isolectin 2**
Non-specific lipid-transfer protein 2G
Avenin-3
Monomeric alpha-amylase inhibitor
Alpha-gliadin
Non-specific lipid-transfer protein (Fragment)
Low-molecular-weight glutenin storage protein
Gliadin/avenin-like seed protein
Thioredoxin H-type
Alpha-gliadin protein
L-1
Alpha-gliadin PSQ2 (Fragment)
Monomeric alpha-amylase inhibitor
